# A Single Nucleotide Variation of *CRS2* Affected the Establishment of Photosynthetic System in Rice

**DOI:** 10.3390/ijms24065796

**Published:** 2023-03-18

**Authors:** Hongwei Chen, Qi Wang, Mingqian Fan, Xijuan Zhang, Pulin Feng, Lin Zhu, Jiayi Wu, Xiaoyi Cheng, Jiayu Wang

**Affiliations:** 1 Key Laboratory of Rice Biology & Genetic Breeding in Northeast China, Ministry of Agriculture and Rural Areas, Rice Research Institute, Shenyang Agricultural University, Shenyang 110866, China; 2 Cultivation and Tillage Institute, Heilongjiang Academy of Agricultural Sciences, Heilongjiang Provincial Engineering Technology Research Center of Crop Cold Damage, Harbin 150086, China

**Keywords:** chloroplast RNA splicing, photosynthesis, chlorophyll fluorescence, map-based cloning, rice (*Oryza sativa* L.)

## Abstract

Chloroplasts are essential sites for plant photosynthesis, and the biogenesis of the photosynthetic complexes involves the interaction of nuclear genes and chloroplast genes. In this study, we identified a rice pale green leaf mutant, *crs2*. The *crs2* mutant showed different degrees of low chlorophyll phenotypes at different growth stages, especially at the seedling stage. Fine mapping and DNA sequencing of *crs2* revealed a single nucleotide substitution (G4120A) in the eighth exons of *CRS2*, causing a G-to-R mutation of the 229th amino acid of CRS2 (G229R). The results of complementation experiments confirmed that this single-base mutation in *crs2* is responsible for the phenotype of the *crs2* mutant. *CRS2* encodes a chloroplast RNA splicing 2 protein localized in the chloroplast. Western blot results revealed an abnormality in the abundance of the photosynthesis-related protein in *crs2*. However, the mutation of *CRS2* leads to the enhancement of antioxidant enzyme activity, which could reduce ROS levels. Meanwhile, with the release of Rubisco activity, the photosynthetic performance of *crs2* was improved. In summary, the G229R mutation in CRS2 causes chloroplast protein abnormalities and affects photosystem performance in rice; the above findings facilitate the elucidation of the physiological mechanism of chloroplast proteins affecting photosynthesis.

## 1. Introduction

A chloroplast is a kind of semiautonomous organelle in plant cells and contains approximately 3000 different proteins [1]. The genome of chloroplasts in higher plants is a circular double-stranded structure without histones and the superhelix structure of the nuclear genome. Chloroplast genes can be divided into three categories according to their function: photosystem genes (*psaA*, *psbA*, *atpA* and *rbcL*, etc.), genesis-related genes (*rrn*, *rpl*, *rps*, *trn*, *rpoA*, *rpoB*, *rpoC1* and *rpoC2*, etc.) and biosynthesis-related genes (*ndhA*-*F*, etc.) [2]. Chloroplast RNA requires RNA-binding proteins for RNA post-transcriptional modifications, such as intron splicing and RNA editing, before translation [3]. Many nuclear-encoded proteins involved in chloroplast RNA post-transcriptional modifications have been identified, such as the pentatricopeptide repeat (PPR) protein, chloroplast RNA splicing and ribosome maturation (CMM) domain-containing protein and ribonuclease III domain-containing protein. In rice, *AL2* encodes a chloroplast splicing facilitator (CRS1) that, by way of its three CRM domains, is involved in the shearing of group I and II introns [4]. The CRM domain contains a conserved “GxxG” motif, which has the ability to bind RNA [5]. *OsPPR6* not only affects *ycf3* RNA splicing efficiency but is also involved in *ndhB* RNA editing. The mutation of *OsPPR6* can lead to abnormal chloroplast development and death due to albinism [6].

In addition to participating in post-transcriptional modification of RNA, RNA-binding proteins also facilitate translation. Ribosomes are protein-synthesizing organelles in living cells [7]. The 70S-type chloroplast ribosome consists of a 50S large subunit and a 30S small subunit. The 30S minor subunit is composed of 24 ribosomal proteins and 1 16S rRNA, while the 50S major subunit is composed of 33 ribosomal proteins and 3 rRNAs: 4.5S, 5S and 23S rRNA [8]. Unlike plastid ribosomal proteins, all four plastid rRNAs are encoded by plastid genes. Plastid ribosomal proteins can be divided into essential and nonessential types, among which the mutation of essential ribosomal proteins can severely hinder photosynthesis and plant growth and development and can even cause death [9]. The mutation of the chloroplast gene *RPS4* in Chinese cabbage significantly reduced the abundance of chloroplast rRNA and chlorophyll content in the leaves [10]. Rice *ASL2* encodes chloroplast ribosomal protein L21; L21 loss-of-function mutant plants exhibit an albino leaf phenotype and die after they reach the four-leaf stage [11]. Recent studies have found that chloroplast translation is generally coupled with transcription. As an RNA-binding protein, PPR53 contains a PPR domain and a carboxyterminal small-mut-related (SMR) domain. The mutation of PPR53 resulted in maize leaf chlorosis and death, loss of *RRN23* and *ndhA* transcripts, and significantly decreased translation efficiency of ndhA mRNA [12]. In addition, PPR proteins, such as PPR10 [13], CRP1 [12] and PGR3 [14] have been shown to affect RNA processing and translation in maize simultaneously. However, all the mutations in these mutants affect RNA processing and translation at the same time and have no direct effect on the activity of RNA-binding proteins on RNA translation.

The photosynthesis system is generally divided into two components: photosystem I (PSI) and photosystem II (PSII). Major proteins of the photosystems, such as psaA and psbA, are encoded by chloroplast genes. Thus, impaired chloroplast translation significantly reduces the chlorophyll content and affects photosynthetic efficiency. In Arabidopsis thaliana, the expression of plastid-specific ribosomal protein genes (PSRPs) was shown to be significantly reduced in related mutants, resulting in defective chloroplast translation, reduced pigment contents and decreased maximum quantum efficiency (Fv/Fm) of PSII [15].

Chloroplasts are the main organelles of plant photosynthesis, and light provides the driving force for photosynthesis [16]. The light-harvesting complex consists of light-harvesting chlorophyll a/b-binding (LHCB) proteins and chlorophyll and transfers the light energy captured by chlorophyll to the reaction center proteins. Plant leaves tend to synthesize excess Chl to build larger light-harvesting antennas, which results in the plant obtaining more light energy than it needs for photosynthesis. Excessive sunlight energy can cause electrons to be transferred to O_2_, which results in the generation of superoxide anion (O_2_^−^) radicals and hydrogen peroxide (H_2_O_2_), which can lead to photooxidative damage. In general, the production rate of reactive oxygen species (ROS) in rice chloroplasts increases when the light intensity surpasses 2000 μmol·m^−2^·s^−1^ [17]. Photosynthetic electron transport chains preferentially undergo cyclic and pseudocyclic electron transport and transfer electrons to O_2_ to form O_2_^−^ [18]. In addition, a high ROS content negatively affects plant growth, leading to oxidative damage of proteins and nucleic acids, as well as membrane lipid peroxidation [19]. Plants employ a series of antioxidant enzymes, such as superoxide dismutase (SOD, EC: 1.15.1.1) and peroxidase (POD), to eliminate excess ROS [20]. Second to photosynthesis, photorespiration has the highest amount of metabolic flux; during photorespiration, plants catalyze ribulose-1,5-bisphosphate (RuBP) to generate glycolate-2-phosphate (2-PG) through the oxygenation activity of the ribulose 1,5-bisphosphate carboxylase and oxygenase (Rubisco, EC: 4.1.1.39) enzyme, and the 2-PG is promptly converted to glycolic acid [21]. Glycolate oxidase (GLO) functions in the peroxisome and can oxidize glycolic acid to glycolic acid and H_2_O_2_. To prevent damage, plants need to convert glycolic acid to 3-phosphoglycerate (PGA) through the photorespiration pathway to return to the Calvin cycle. Photorespiration not only removes toxic intermediates but also acts as an important regulatory mechanism of energy dissipation, effectively metabolizing reducing power and preventing photoinhibition [22]. Plants can also dissipate excess sunlight energy in the form of heat through nonphotochemical quenching (NPQ) to prevent high light stress.

In this study, the *chloroplast RNA splicing 2* (*crs2*) mutant was obtained via ethyl methane sulfonate (EMS)-treated *Oryza sativa* japonica cv. Shennong 9816 (SN9816). The leaves of the mutants showed a pale green phenotype, and the chlorophyll content was reduced. Map-based cloning and genetic complementation tests revealed that the *crs2* mutation was responsible for the abnormal chlorophyll content phenotype. Western blot and qPCR analyses indicated that *CRS2* affects the expression of chloroplast rRNA and the relative abundance of major photosynthetic proteins. In addition, the *crs2* mutant experienced less thermal dissipation and higher electron transfer efficiency, leading to an increase in the net assimilation rate (An). In conclusion, our study provides a new way in which the regulatory effects of *CRS2* on chloroplast development and photosystem performance in rice are revealed.

## 2. Results

### 2.1. Phenotype of Mutant crs2

**Figure 1:**
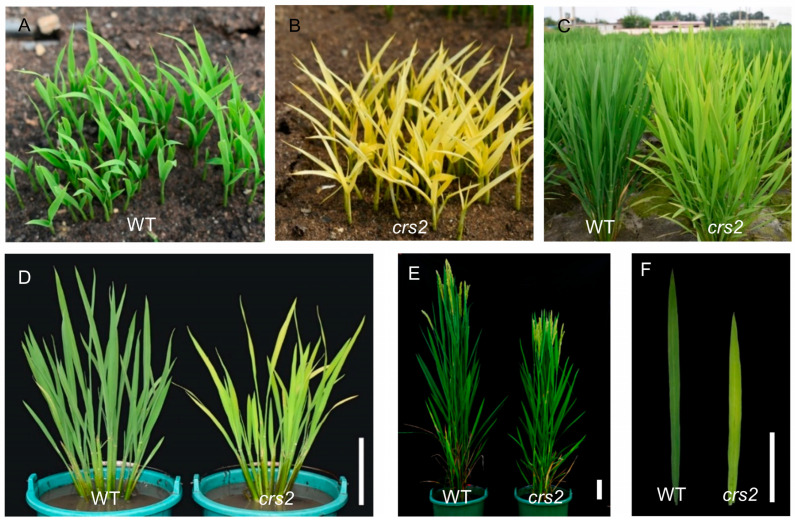
Phenotype and main agronomic traits of the WT and *crs2*. (**A**,**B**) Phenotypes of the WT and *crs2* at the seedling stage (25 DAS). (**C**,**D**) Phenotypes of the WT and *crs2* at the tillering stage (75 DAS). (**E**) Phenotypes of the WT and *crs2* at the full-heading stage (110 DAS). (**F**) Flag leaves of the WT and *crs2* at the heading stage. Bar = 10 cm.

**Figure 2:**
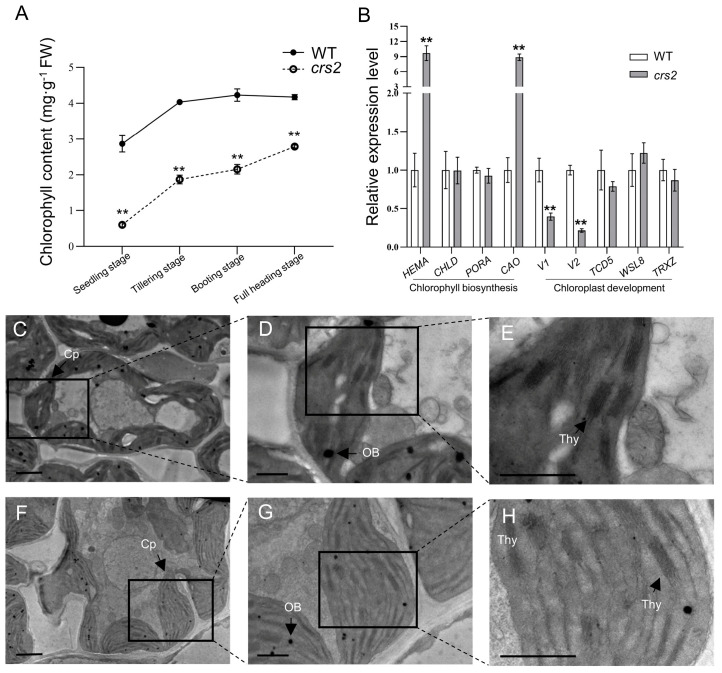
Analysis of chlorophyll biosynthesis and chloroplast development in the WT and *crs2*. (**A**) Chlorophyll content in the WT and *crs2* at different stages. (**B**) Relative expression levels of genes related to chlorophyll biosynthesis and chloroplast development. *HEMA* (*Os10g0502400*); *CHLD* (*Os03g0811100*); *PORA* (*Os04g0678700*); *CAO* (*Os10g0567400*); *V1* (*Os03g0656900*); *V2* (*Os03g0320900*), *virescent-2*; *TCD5* (*Os05g0411200*); *WSL8* (*Os05g0430200*); *TRXZ* (*Os08g0378900*). (**C**–**H**) Chloroplast ultrastructure images in the WT (**C**–**E**) and *crs2* (**F**–**H**) plants. Bar = 1 μm. Cp, chloroplast; Thy, thylakoid lamella; OB, osmophilic body. Mean ± SD. ** extremely significance at *p* < 0.01 (Student’s *t*-test).

The *crs2* mutant was obtained from an EMS mutant pool of the japonica rice cultivar SN9816. Under field conditions, the mutant *crs2* leaves showed different degrees of pale green phenotypes. The difference in plant color was most obvious at the seedling stage and then gradually diminished (Figure 1A–F). The chlorophyll content of *crs2* decreased by 79.0% compared with the WT at the seedling stage, while only a 22.2% decrease could be observed at the full-heading stage (Figure 2A). The results of the chlorophyll content were consistent with the phenotypes of the materials in the field, and they indicated that leaf color changes of the *crs2* mutants were associated with a low photosynthetic pigment content.

Although the chlorophyll content of *crs2* decreased, the relative expression levels of *CHLD* and *PORA*, which are related to chlorophyll biosynthesis, did not change significantly. Moreover, the expression levels of *HEMA* and *CAO* increased significantly (Figure 2B). Among those of chloroplast development-related genes, the expression levels of *V1* and *V2* in *crs2* were significantly lower than those in the WT, while the expression levels of *TCD5*, *WSL8* and *TRXZ* were not significantly different between the WT and *crs2* (Figure 2B).

However, the results of TEM showed that the mesophyll cells of the WT and *crs2* plants presented normal chloroplasts with well-developed stacked grana, thylakoid lamellae and thylakoid membranes at the tillering stage (Figure 2C–H). There were no significant differences in the average thickness of the granum stacks or the number of granum stacks per chloroplast between the WT and *crs2* (Figure S1). Although the expression of the V1 and V2 genes in the *crs2* mutant decreased, this probably did not affect chloroplast development.

### 2.2. Map-Based Cloning of CRS2 and Complementation Analysis

**Figure 3:**
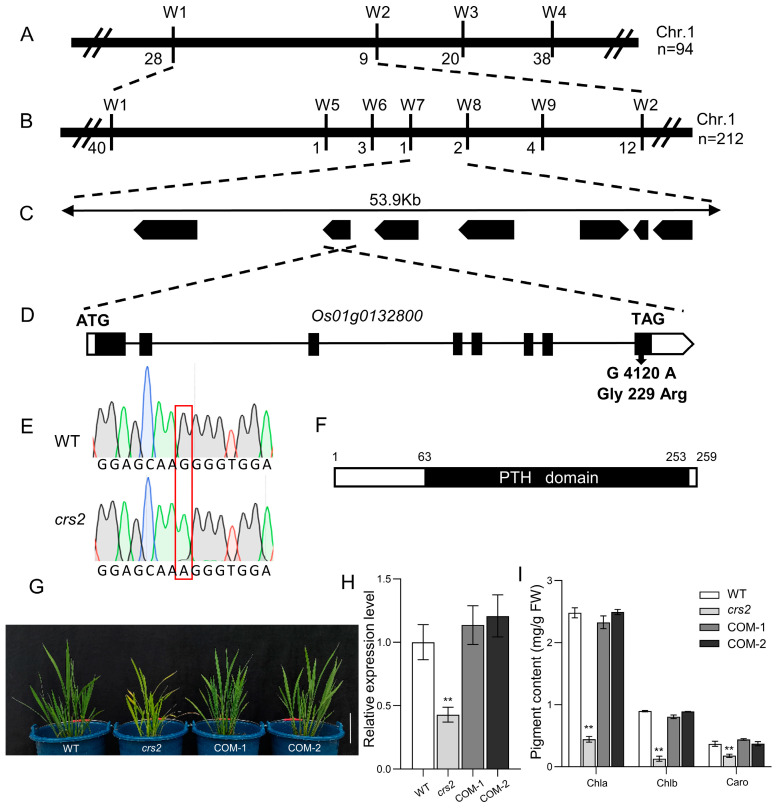
Map-based cloning of *CRS2* and transgenic complementation of the *crs2* mutant. (**A**) *CRS2* was mapped between markers W1 and W2 on chromosome 1. (**B**) Fine mapping of *CRS2*. The *CRS2* locus was further mapped to a 53.9 kb region between markers W7 and W8. (**C**) Seven putative open-reading frames (ORFs) are located in this region. (**D**) Structure of the candidate gene *Os01g0132800*. The candidate gene *Os01g0132800* comprises eight exons and seven introns, in which a single nucleotide G-to-A substitution occurs at position 4120 bp from the ATG start codon. The black rectangle represents the exon. (**E**) DNA sequencing peaks of the WT and *crs2*. (**F**) Domain analysis of the CRS2 protein. The CRS2 protein contains a PTH domain. (**G**) Functional complementation of *CRS2* restored the phenotypes of the *crs2*. Bar = 10 cm. (**H**) Expression levels of CRS2 in the WT, *crs2*, COM-1 and COM-2 cells. (**I**) Content of pigment in the WT, *crs2*, COM-1 and COM-2. Mean ± SD. ** extremely significance at *p* < 0.01 (Student’s *t*-test).

For genetic analysis, the *crs2* mutant was crossed with the wild type, and the obtained F_1_ generation hybrids showed normal green leaves. Among all 1137 F_2_ population individual plants, 861 showed a normal phenotype, and 276 showed a mutant phenotype (Table S1), with a 3:1 ratio of trait isolation (χ^2^ = 0.319). These results indicate that the abnormal pigment phenotype of *crs2* is attributable to the inheritance of a single recessive gene. To determine the physical location of the mutant gene, we constructed an F_2_ population with *crs2* and Kasalath as parents and performed a chromosomal linkage analysis on 94 individual plants with a mutant phenotype (Figure 3A). After expanding the mapping population to 212, *crs2* was precisely mapped to a 53.9 kb interval between W7 and W8 (Figure 3B), in which there are seven open-reading frames (ORFs) (Figure 3C). Then, these seven ORFs were sequenced, and only a single-nucleotide mutation (G4120A) was found at position 4120 bp from the ATG start codon in *Os01g0132800*, which resulted in the amino acid substitution of Gly229 to Arg229 (G229R) (Figure 3D–F). To confirm whether the mutation of *Os01g0132800* resulted in a mutant phenotype, we transferred the complementary vector containing the *CRS2* genome sequence into the *crs2* mutant and obtained a total of 19 positive transgenic plants, all of whose mutant phenotype was rescued (Figure 3G). Pigment contents and gene expression levels were measured, and all of these parameters of the transgenic plants were restored to the WT levels (Figure 3H,I). Thus, these results confirmed that *Os01g0132800* corresponded to the *CRS2* gene and that the single-nucleotide substitution of *Os01g0132800* is responsible for the abnormal chloroplast translation of the *crs2* mutant. The *CRS2* gene comprises eight exons and encodes a chloroplast RNA splicing 2 protein composed of 259 amino acids (Figure 3D). Only one peptidyl-tRNA hydrolase (PTH) domain was found via conserved domain analysis (Figure 3F).

### 2.3. Expression Pattern and Subcellular Localization of CRS2

**Figure 4:**
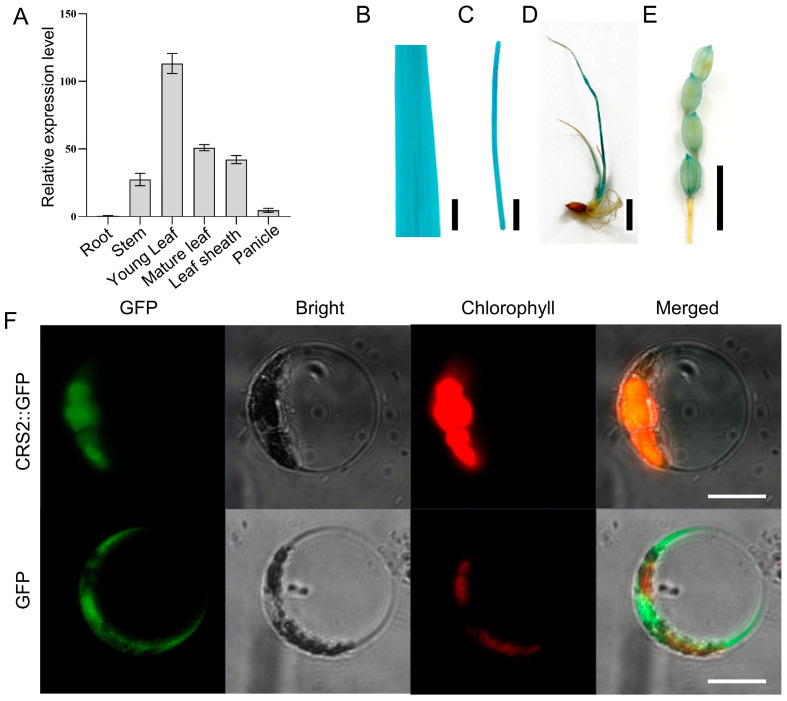
Tissue expression pattern of *CRS2* and subcellular localization of the CRS2-GFP in rice protoplasts. (**A**) Relative expression levels of *CRS2* in various tissues. (**B**–**E**) β-glucuronidase (GUS) staining of leaves (**B**), stems (**C**), young leaves (**D**) and panicles (**E**) of transgenic rice expressing pCRS2::GUS. Bar = 1 cm. (**F**) Subcellular localization of the CRS2 protein. Rice protoplast transformed with p35S::CRS2::GFP and transformed with p35S::GFP as a control. Bar = 10 μm.

To explore the expression pattern of *CRS2* in WT plants, qRT–PCR analyses were performed on the roots, stems, leaves, leaf sheaths and panicles. In general, the *CRS2* gene was expressed in all the aboveground parts of the rice plants. The highest expression part was found in the young leaves, followed by the mature leaves (Figure 4A). Furthermore, a histochemical staining analysis of CRS2-GUS transgenic plants was conducted to confirm the expression pattern of *CRS2*. The *CRS2* gene was expressed in the stems, leaves, leaf sheaths and panicles and was expressed mainly in the leaves, but no *CRS2* expression was detected in the roots (Figure 4B–E). These results were consistent with the qRT–PCR results.

We constructed a subcellular localization vector with pCAMBIA1300-EGFP, which uses the CaMV 35S promoter to drive the expression of the CRS2-GFP fusion protein. After transient expression in rice protoplasts, the green fluorescence signal of the CRS2-GFP fusion protein coincided with that of the chloroplast, and empty GFP was present in the cytoplasm (Figure 4F). These results confirm that CRS2 was localized in the chloroplasts and was mainly expressed in green tissues.

### 2.4. Abnormal Chloroplast Proteins in crs2

**Figure 5:**
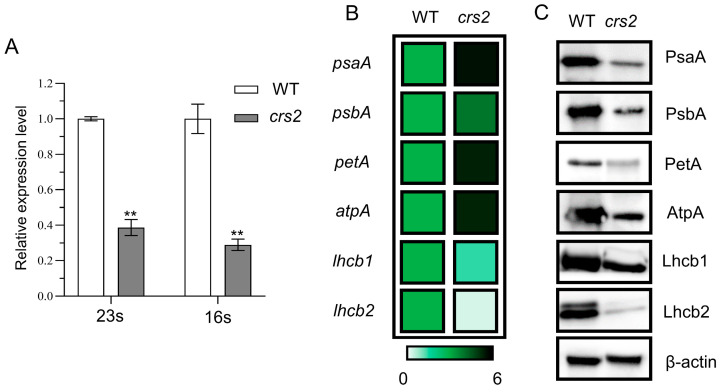
Variations in photosynthesis-related gene expression levels. (**A**) Expression levels of 16S and 23S in the WT and *crs2*. (**B**) Relative expression levels of photosynthesis-related genes in the WT and *crs2*. (**C**) Western blots of proteins related to photosynthesis in the WT and *crs2*. β-actin was used as an internal control. Mean ± SD. ** extremely significance at *p* < 0.01 (Student’s *t*-test).

Analysis of chloroplast intron splicing showed that *crs2* intron splicing was not affected (Figure S2), and the expression levels of 23S and 16S rRNA in the chloroplasts were significantly lower in *crs2* than in the WT (Figure 5A). The qRT–PCR results showed that the expression levels of *psaA*, *psbA*, *petA* and *atpA* encoded by the chloroplast genome in *crs2* were significantly higher than those in the WT, but the expression levels of *lhcb1* and *lhcb2* encoded by nuclear genome were significantly lower (Figure 5B). Western blot analysis revealed that the abundance of the PsaA, PsbA, PetA, AtpA, Lhcb1 and Lhcb2 proteins in *crs2* significantly decreased (Figure 5C). 

### 2.5. Analysis of Chlorophyll Fluorescence

**Figure 6:**
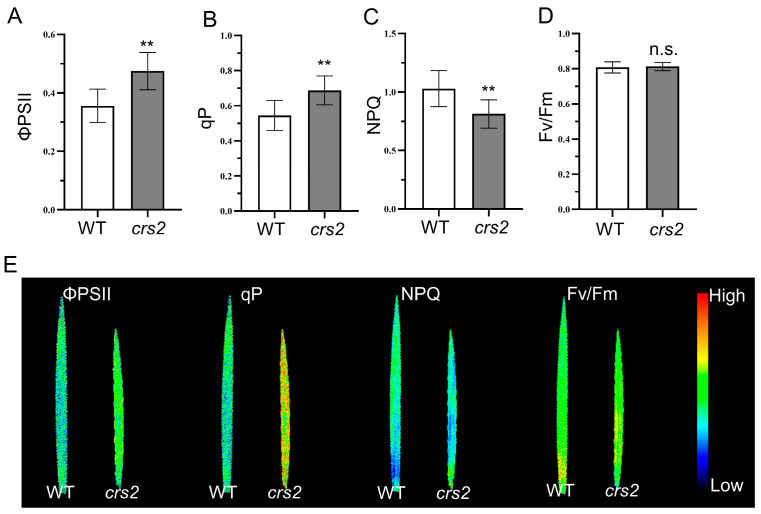
Fluorescence parameters of the WT and *crs2*. (**A**–**D**) Variations in the ΦPSII (**A**), qP (**B**), NPQ (**C**) and Fv/Fm (**D**). (**E**) Chlorophyll fluorescence image. Mean ± SD. ** extremely significance at *p* < 0.01, n.s., no significant (Student’s *t*-test).

The reduction in chlorophyll content in the *crs2* mutants not only resulted in the abnormal pigment phenotype but also affected the absorption and utilization of light energy in *crs2*. The chlorophyll fluorescence parameters under steady-state photosynthesis indicated that, compared with that of the WT, the Fv/Fm of *crs2* did not change significantly; however, the quantum yield of PSII photochemistry (ΦPSII) and the photochemical quenching coefficient (qP) increased by 33.4 and 26.2%, respectively, and the NPQ decreased by 15.2% (Figure 6A–D). The chlorophyll fluorescence imaging results were also consistent with the above results (Figure 6E). The ΦPSII and qP of *crs2* are significantly higher than that of the WT, while the NPQ is significantly decreased. The Fv/Fm images showed no significant difference between the WT and *crs2*. Taken together, these results indicated that the decrease in chlorophyll content in the mutant did not affect the photosynthesis potential at the full head stage. The light energy absorbed by the pigment in the antenna of the PSII reaction center was used more for photosynthetic electron transport, which might contribute to the release of the activity of PSII in the reaction center.

### 2.6. High Light Adaptability Comparison between WT and crs2

**Figure 7:**
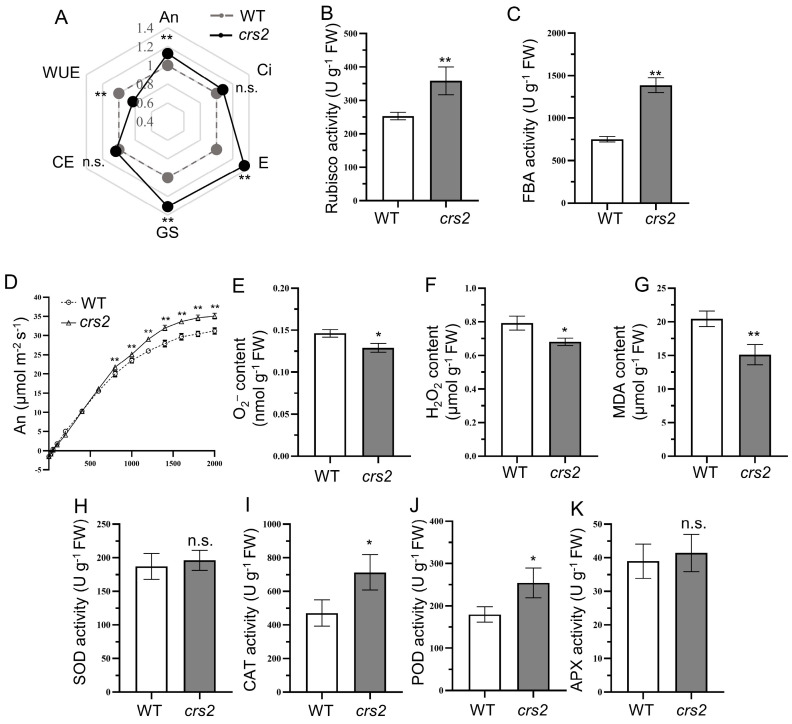
Variations in photosynthesis-related gas exchange parameters and enzyme activity in the WT and *crs2*. (**A**) Radar map of photosynthesis-related indicators, including net assimilation rate (An), intercellular CO_2_ concentration (Ci), transpiration rate (E), stomatal conductance (GS), carboxylation efficiency (CE) and the water use efficiency (WUE) per unit area of leaf surface area of the WT and *crs2*. (**B**) Rubisco activity. (**C**) FBA activity. (**D**) An of the WT and *crs2* in response to light intensity. (**E**) Superoxide anion (O_2_^−^) content. (**F**) H_2_O_2_ content. (**G**) MDA content. (**H**) SOD activity. (**I**) CAT activity. (**J**) POD activity. (**K**) APX activity. Mean ± SD. * significance at *p* < 0.05, ** extremely significance at *p* < 0.01, n.s., no significant (Student’s *t*-test).

Compared with those of the WT, the chlorophyll content and photosynthesis-related protein abundance of *crs2* decreased significantly, but the An, intercellular CO_2_ concentration (Ci), stomatal conductance (gs) and transpiration rate (E) increased by 12.4, 7.8, 30.9 and 34.2%, respectively. In addition, the water use efficiency (WUE) decreased significantly, and there was no significant difference in carboxylation efficiency (CE) (Figure 7A). In *crs2*, the activity of Rubisco and FBA enzymes were significantly increased and were 41.8 and 84.5% higher than those in the WT, respectively (Figure 7B,C). The light response curve showed that the An of *crs2* was significantly higher than that of the WT when the PPFD was ≥800 μmol·m^−2^·s^−1^ (Figure 7D). For the antioxidant enzymes, the CAT and POD activities in *crs2* significantly increased (Figure 7I,J), while the SOD and APX activities were not significantly different (Figure 7H,K). The increase in antioxidant enzyme activity in the leaves of the *crs2* mutant indicated that the mutant had a high antioxidant capacity and could reduce the ROS content in mesophyll cells, and compared with the WT, the mutant had a better protective effect on mesophyll cells and chloroplasts. In addition, compared with those in the WT, the content of O_2_^−^, H_2_O_2_ and MDA in the *crs2* mutant were reduced by 11.8, 14.2 and 26.1%, respectively (Figure 7E–G). Therefore, *crs2* can withstand ROS stress caused by high light intensity.

## 3. Discussion

### 3.1. The G229R Mutation in CRS2 Is Responsible for the Decreased Chlorophyll Content

In this study, the *crs2* mutant was obtained by EMS mutagenesis, whose leaf was pale green. Although the chlorophyll content of *crs2* was decreased, the expression level of genes involved in chlorophyll synthesis was significantly increased, indicating that the abnormal chlorophyll content of *crs2* was not caused by the obstruction of chlorophyll synthesis. Map-based cloning and gene complementation validation indicated that a single nucleotide substitution of *crs2* caused the abnormal chlorophyll content of the mutant. *CRS2* encodes a chloroplast RNA splicing 2 protein, which is one of the best-characterized chloroplast RNA splicing factors [23]. Jenkins et al., identified a T-DNA insertion mutant *zmcrs2* in maize, whose group II intron splicing was abnormal, and the albino leaf phenotype appeared [23]. CRS2 evolved from a peptidyl tRNA hydrolase, but a neofunctionalization resulted in it becoming one of the most important and conserved splicing factors in plant chloroplasts. Due to the lack of RNA binding ability, CRS2 needs to interact with CAF1 and CAF2 and form a splicing complex to participate in RNA splicing [24]. Both CAF1 and CAF2 are CRM domain-containing proteins, in which the CRM domain contains the conserved GxxG sequence and has the ability of RNA binding [5]. Studies on rice *crs2* mutants found that CRS2 is also involved in the splicing of a group I intron and chloroplast rRNA maturation [25]. Null mutants of *CRS2* are seedling-lethal in maize rice, but the G229R mutation seems to be hypomorphic and has a normal mRNA splicing length and did not show seedling albino lethal phenotypes. In the present study, the G229R mutations led to impaired biogenesis of chloroplast ribosomes and abnormal chloroplast protein abundance. Chlorophyll can be combined with light-harvesting chlorophyll a/b-binding proteins to form light-trapping pigment-protein complexes that capture light energy and rapidly transfer it to the reaction centers to trigger photochemical reactions [26]. The abundance of light-harvesting a/b-binding proteins in *crs2* decreases along with that of PsaA and PsbA, thus leading to a decrease in chlorophyll content.

### 3.2. The crs2 Mutant Shows Higher Photosynthetic Efficiency

Chlorophyll fluorescence is an important photosynthesis parameter that reflects the absorption and utilization of light energy by PSII [27]. It is generally believed that the decrease of chlorophyll content may impair the photosynthetic performance, but the results of photosynthetic performance studies in some related mutants are different. For example, the mutation of *YGL53* seriously affected chlorophyll synthesis in rice, but the electron transfer ability of the *ygl53* mutant was significantly improved, which activated the activity of PSII [28]. Therefore, the chlorophyll fluorescence parameters were further detected in this study. The Fv/Fm value indicates PSII’s maximum potential for photosynthetic efficiency, while there was no significant difference observed between the WT and the mutant. ΦPSII represents the actual photochemical efficiency of PSII, and qP reflected the amount of energy consumed and the openness of the PSII reaction center. In this study, qP and ΦPSII were significantly increased in *crs2* mutant, while it could be seen that NPQ was significantly decreased. NPQ represents the energy dissipated as heat in a non-photochemical quenching process. Some studies have demonstrated a negative correlation between ΦPSII and NPQ [29]. Similar to *ygl53*, the lower NPQ of the *crs2* mutant indicated that, by reducing heat dissipation, the mutant may increase its electron transfer rate and light energy use efficiency. In addition, Rubisco and FBA are important rate-limiting enzymes in plant photosynthesis, and the improvement of the activities of Rubisco and FBA in *crs2* can accelerate the rate of photosynthesis, thus improving the light energy using efficiency [30,31]. The results in the present study indicated that the *crs2* mutant achieved a higher ΦPSII by increasing the qP to make better use of light energy, along with the reduction of non-photochemical quenching.

### 3.3. High Protective Enzyme Activity Is the Guarantee of crs2 to Cope with the Intensity of the High Light

It is widely believed that plant leaves tend to synthesize excess amounts of chlorophyll to build larger antennas that capture and absorb more light energy than that demanded by photosynthesis [32]. The excess light energy absorbed needs to be quenched by a variety of light energy dissipation mechanisms to avoid ROS stress and photoinhibition, avoiding the reduction in light energy conversion efficiency [33,34]. Both the chlorophyll content and the abundance of light-trapping antenna proteins in the *crs2* mutant decreased, resulting in a smaller size of pigment antennas in the reaction center. Nonetheless, the measurement of photosynthetic indices showed that although the size of the pigment antenna of *crs2* decreased, the net photosynthesis rate of *crs2* increased significantly. This phenomenon was also observed in indica rice *pe-1* mutants, which presented a decreased chlorophyll content but enhanced photosynthesis [35]. Therefore, the smaller pigment antenna of the *crs2* mutant could still capture the light energy required for photosynthesis. The intact chloroplast structure indicated that *crs2* had normal photosystem function. The increase in Rubisco activity ensured that the *crs2* mutant had a high capacity for CO_2_ fixation. As the rate-limiting enzyme of photosynthesis, Rubisco is involved in the first step of catalytic photosynthetic carbon assimilation, and Rubisco activity has a positive effect on the CO_2_ assimilation rate of plants [36]. There are three pathways of photosynthetic electron transport in higher plants: noncyclic electron transport, cyclic electron transport and pseudocyclic electron transport [37]. In our study, the efficiency of electron transport in the *crs2* mutant is improved through optimization of the use of light energy, and the photosystems capture more energy flowing as part of noncyclic electron transport. Due to the increase in ROS scavenging activity, the O_2_^−^ and H_2_O_2_ content in *crs2* decreased substantially. Normal conditions lead to a balance between ROS generation and scavenging, which is harmless to plants [38]. However, ROS stress can lead to lipid peroxidation and MDA production in plants, and MDA content is an important index for reflecting the degree of oxidative damage to cells [39]. The smaller size of the light-capturing antenna and higher light energy use efficiency of *crs2* can reduce the production of ROS, consequently reducing the level of membrane lipid peroxidation and thus reducing the MDA content. Moreover, the increased activities of CAT and POD also reflected the enhanced ROS-scavenging ability of *crs2*. On the other hand, the reduction in ROS levels and the enhancement of the scavenger system are important for *crs2* to prevent ROS stress and photoinhibition under high light intensity. The increase in CAT activity could also enhance the activity of Rubisco [40] and further improve the carbon assimilation ability of *crs2*.

## 4. Materials and Methods

### 4.1. Plant Materials and Growth Conditions

To map *crs2*, we constructed an F_2_ population derived from a cross of the *crs2* mutant and the indica rice variety Kasalath. The seedlings of the materials were cultivated using thermal preservation and dry seedling–nursery technology. The seedlings were sown in mid-April and transplanted 30 days later. All the plants were transplanted under natural conditions into a paddy field of the Rice Research Institute, Shenyang Agricultural University (41° N, 123° E).

### 4.2. Measurement of Chlorophyll Content

Fresh leaf tissues (0.2 g) were soaked in 5 mL of 95% ethanol for 48 h in darkness. The absorbance values of the supernatant at 470, 649 and 665 nm were measured using a Hitachi U5100 spectrophotometer (Hitachi, Tokyo, Japan), and the pigment content was calculated using the method of Arnon [41]. Three biological replicates were performed for each sample.

### 4.3. Transmission Electron Microscopy (TEM)

WT and *crs2* fresh leaves were taken and cut into 0.25 cm^2^ pieces. The cells were fixed in 3% glutaraldehyde solution and 1% OsO_4_ successively and then embedded in resin after being subjected to a dehydration gradient. Then, the leaves were stained with uranyl acetate and alkaline lead citrate and observed under a transmission electron microscope (HITACHI HT7800, Hitachi); the magnifications used for observation were 1.5, 5.0 and 12.0 k×.

### 4.4. Determination of ROS-Related Physiological Indices

Fresh leaves of WT and *crs2* plants were taken at the tillering stage. The main veins were removed, and 0.1 g samples were taken for subsequent determination. Rubisco activity (BC0440), FBA activity (BC2270), O_2_^−^ content (BC1290), H_2_O_2_ content (BC3590) and malondialdehyde (MDA) content (BC0020) were determined using a kit (Beijing Solarbio Science & Technology Co., Ltd., Beijing, China). The activities of SOD (A001-3-2), POD (A084-3-1), catalase (CAT) (A007-1-1) and ascorbate peroxidase (APX, EC: 1.11.1.11) (A123-1-1) were measured using kits obtained from the Nanjing Jiancheng Research Institute. All the protocols were followed according to the manufacturer’s instructions. A UV spectrophotometer was used to measure light absorption value and calculate each index. Three biological replicates were measured for each sample.

### 4.5. Measurement of Photosynthetic Gas Exchange and Chlorophyll Fluorescence Parameters

Photosynthesis and fluorescence parameters were determined on sunny day at full heading stage. Gas exchange parameters of flag leaves were measured using a portable photosynthesis system (CIRAS 3); the photosynthetic photon flux density (PPFD) was set to 1200 μmol·m^−2^·s^−1^, and the CO_2_ concentration was set to 390 ppm and stabilized using a CO_2_ injector system. To determine the response of plants to different light intensities, the net assimilation rates of WT and *crs2* were measured under 0, 50, 100, 200, 400, 600, 800, 1000, 1200, 1400, 1600, 1800 and 2000 μmol·m^−2^·s^−1^ actinic light. After dark treatment with leaf clips for 30 min, an FMS-2 pulse-modulated fluorometer (Hansatech, Norfolk, UK) was used to measure modulated chlorophyll fluorescence, and the chlorophyll fluorescence was observed using a FluorCam 7 chlorophyll fluorescence imaging system (Photon Systems Instruments, Brno, Czech Republic). Five independent individuals were measured for each sample.

### 4.6. Map-Based Cloning and Complementation of CRS2

We obtained 212 plants with mutant epitopes from the constructed F_2_ population and extracted DNA for map-based cloning of *CRS2*. The genomic sequences of Nipponbare (japonica) and 93-11 (indica) were downloaded from the website of the Rice Genome Annotation Project and used to develop simple sequence repeat (SSR) and insertion–deletion (Indel) markers.

To complement the *crs2* mutation, the 6734 bp genome sequence was amplified from DNA of wild-type SN9816 leaves and cloned into the binary expression vector pCAMBIA1300 to construct a pCAMBIA1300-CRS2 vector. The recombinant vector was subsequently transferred into mutant callus by Agrobacterium tumefaciens EHA105. The sequences of all the primers used are listed in Supplementary Table S2.

### 4.7. RNA Extraction and Quantitative Real-Time PCR (qRT–PCR) Analysis

Total RNA was extracted from tissue using a MiniBEST Plant RNA Extraction Kit (9769S, Takara Bio, Inc., Kusatsu, Japan) according to the manufacturer’s instructions. The cDNA was reverse-transcribed by using a PrimeScript RT Reagent Kit (RR047Q, Takara Bio, Inc.). qRT–PCR was subsequently performed using SYBR Premix Ex Taq II (RR820A, Takara Bio, Inc.) in an ABI 7500 Real-Time Fluorescent PCR System according to the manufacturer’s instructions. The real-time PCR experiments were repeated for three biological replicates.

For chloroplast gene intron splicing analysis, the group I and group II introns of the WT and *crs2* genes were amplified and isolated via 1% agarose gel electrophoresis. The primers used for intron splicing and qRT–PCR are listed in Supplementary Table S2. Three independent experiments were repeated for each sample.

### 4.8. Histochemical β-Glucuronidase (GUS) Assays

To conduct active staining of the transgenic plants, a 2123 bp region of the *CRS2* promoter was amplified from SN9816 genomic DNA using PCR and ligated into a pCAMBIA1305 vector such that it drove the expression of a GUS reporter gene. All the vectors were transformed into WT calli by Agrobacterium (EHA105)-mediated transformation. For GUS staining, we used excised tissues of independent T1 transgenic plants. The material was added to X-GluC buffer and incubated at 37 °C for 2 h. Afterward, the material was decolorized by soaking it in ethanol and imaged using a digital camera. The primers used for vector construction are listed in Supplementary Table S2.

### 4.9. Subcellular Localization of the CRS2 Protein

The full-length coding region of *CRS2* was amplified using PCR and cloned into a pCAMBIA1300-EGFP vector to generate a p35S-CRS2-GFP fusion construct. The fusion construct was subsequently introduced into and transiently expressed in rice protoplasts. GFP signals were detected using a laser scanning confocal microscope (Zeiss, Oberkochen, Germany). The related primers used are listed in Supplementary Table S2.

### 4.10. Protein Extraction and Western Blotting

Total protein was extracted from rice leaf tissue via a plant protein extraction kit (BC3720, Beijing Solarbio Science & Technology Co., Ltd.), and the protein concentration was determined using the bicinchoninic acid assay (BCA) method. The total protein concentration of WT and *crs2* was quantitatively consistent and denatured in boiling water bath for 5 min. The target protein was separated using SDS–PAGE and blotted onto a polyvinylidene difluoride (PVDF) membrane. After incubation with anti-PsaA (AS06 172, Agrisera, Vännäs, Sweden), anti-PsbA (AS05 084), anti-PetA (AS08 306) and anti-AtpA (AS08 304) antibodies, color development was performed using ECL Western Blotting Substrate (PE0010, Beijing Solarbio Science & Technology Co., Ltd.). The anti-β-actin antibody K800001M was used as an internal control.

### 4.11. Statistical Analysis

All of the data were statistically analyzed using Microsoft Excel 2016 (Microsoft Corporation, Washington, DC, USA) and SPSS 22 (SPSS Inc., Chicago, IL, USA). Student’s *t*-test was conducted to compare WT and *crs2*, and * and ** represent significant differences at the 0.05 and 0.01 levels, respectively. GraphPad Prism 8 (GraphPad Software, Inc., San Diego, CA, USA) and Microsoft Excel were used to construct graphs.

## 5. Conclusions

This study indicated that *CRS2* in rice encodes a chloroplast RNA splicing 2 protein located in the chloroplast. The missense mutation of *crs2* resulted in an abnormality in the abundance of chloroplast photosystem complex proteins, thereby causing a decrease in chlorophyll contents. However, this phenomenon did not affect the chloroplast ultrastructure, and the photosynthesis of *crs2* mutants was maintained at normal levels. Taken together, the *crs2* mutation causes abnormalities in rice chloroplast proteins and affects photosystem performance. This research helps to elucidate the physiological mechanism of chloroplast proteins affecting photosynthesis.

## Data Availability

All data generated or analyzed during this study are included in this published article.

## Supplementary Materials

The following supporting information can be downloaded at: https://www.mdpi.com/article/10.3390/ijms24065796/s1.
