# Supplementary material for: A Single Nucleotide Variation of CRS2 Affected the Establishment of Photosynthetic System in Rice
Source: Int J Mol Sci. 2023 Mar 18;24(6):5796. doi: 10.3390/ijms24065796 (PMC10054620; doi:10.3390/ijms24065796)
Supplement: Supplementary file 1 [file ijms-24-05796-s001.zip › ijms-2265350-supplementary.pdf]

Supplementary Materials

1 Supplementary Figures

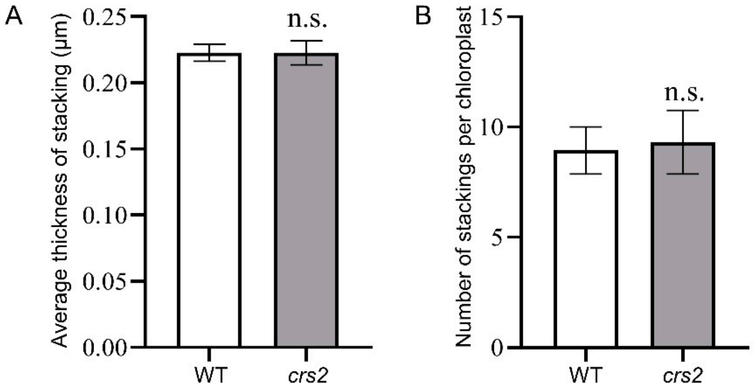

Supplementary Figure S1. (A) Average thickness of stacking in the WT and *crs2*. (B) Number of stackings per chloroplast in the WT and *crs2*. n.s., no significant.

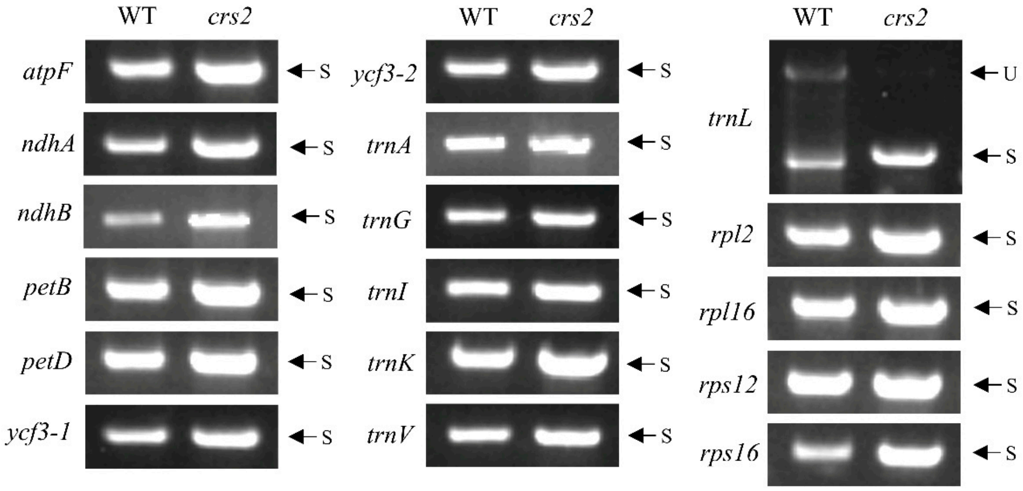

Supplementary Figure S2. Chloroplast gene intron splicing analysis in the WT and *crs2*.

2 Supplementary Table

Supplementary Table S1. Genetic analysis of *crs2*.

Cross Combination	Normal green	Yellow green	Total	χ^2 (3:1)	P-value
crs2×SN9816	861	276	1137	0.319	0.572

Supplementary Table S2. All primers used in the study

Primers	Sequence
W1-F	CTCGCCTCAACAAATCAGGT
W1-R	TCGTCTGCACTGGTTTTGAC
W2-F	ACAAGACGAGGTAACACGCAAGC
W2-R	GCGAAGGATCAACGATGATATGG
W3-F	TCCCACTTGCAGACGTAGGTAGG
W3-R	CACTGATCTGATGCAACTGTTTGG
W4-F	ATCCCAACTCTAAGCCACCC
W4-R	CTACCCGTCACCAACTCACC
W5-F	CCTCAACTGCAATTCTGCAA
W5-R	AGCAACCAATACCACGAAGG
W6-F	GTTGAGCTGAGAGGTGATGAACC
W6-R	CTTCTGTTCTTGGCTTGGTTTCC
W7-F	CTGATGAATGCCGGTAGGTT
W7-R	GTGGCAATCTGAGAGCGTTT
W8-F	CAGCAAGCTGATCTGCAACTCC
W8-R	CAGATACCAACTTTACTTGCCACTCC
W9-F	TACCGCGGATATGGGAATAA
W9-R	CGCATGTCCTCCATTTCTTT
atpF-F	TTTTAGCTCACTGGCCATCC
atpF-R	TTCATCGCCCTTTGTTTTTC
petB-F	ATTCAGACCTCGCAACCAGA
petB-R	GTTGGCCTCGGTCATTATGT
ycf3-1-F	TGATAAGACCTTCTCAATTGTAGCC
ycf3-1-R	GTGTGTATAAGGCCTATGTTATAGAGT
ycf3-2-F	AGAGCATACAAAGGCTTTGGAAT
ycf3-2-R	TTCAACCAGTTCTGTGCTTCAATATA
rpl2-F	ATCGATTTTCGACGGAATCA
rpl2-R	GGACCTCCCCAGATGGTAAT
petD-F	CCATGAAGAGGCTCCGTAAG
petD-R	ATCATTGACGGCTCGAGAAC
rpl16-F	TGCTTCGTATTGTCGAGATCC
rpl16-R	TCGTGTCATTGCTCTTCGTC

ndhA-F	TCCTTTGGGATATCGCTTTG
ndhA-R	TCTTCCTCCGCTTCTGGTAA
ndhB-F	TGGGGCAAGCTCTTCTATTC
ndhB-R	AGCAAGGAGATTCCCCAATA
rps16-F	TTCAAGGAAATAGAGAATAG
rps16-R	AACATGTGGTAGAAAGCAA
trnK-F	TATGGGTTGCCCGGGACTCG
trnK-R	GGTTGCTAACTCAATGGTAG
trnA-F	GGGGATATAGCTCAGTTGGT
trnA-R	TGGAGATAAGCGGACTCGAA
trnI-F	TGGGCCATCCTGGACTTGA
trnI-R	AGCTCAGTGGTAGAGCGCG
trnG-F	TCGTTAGCTTGGAAGGCTAG
trnG-R	GCGGGTATAGTTTAGTGGTA
trnV-F	TAGGGCTATACGGATTCGAA
trnV-R	AGGGCTATAGCTCAGTTCGG
rps12-F	ACTATCAACCCCAAAAAACC
rps12-R	TTTGGCTTTTTGACCCCAT
trnL-F	GGATATGGCGAAATCGGTAGAC
trnL-R	ACCCTCACGACTTAGAAAGTCGAC
HEMA1-F	CGCTATTTCTGATGCTATGGGT
HEMA1-R	TCTTGGGTGATGATTGTTTGG
CHLD-F	GCTTGCAGAAAGCTACACAAGC
CHLD-R	AGGCCGTGAGCTAAAGGAGC
PORA-F	TGTACTGGAGCTGGAACAACAA
PORA-R	GAGCACAGCAAAATCCTAGACG
CAO-F	GATCCATACCCGATCGACAT
CAO-R	CGAGAGACATCCGGTAGAGC
V1-F	TGGAGGTCGGGACAGAGGA
V1-R	CGAGGAGCACCACCATCAC
V2-F	CGACAAGCAGAGCGAAGCG
V2-R	AGGTTGCTGCTCCTTGAATGT
TCD5-F	TAAAAGGAGTTGTCGTGTCTGA
TCD5-R	AAGCATACTGTATGTGTCACGA
WSL8-F	AACCTCTCAGACTGATGGAAAG
WSL8-R	AGTCGTAAATGCTGATCTCCAT
TRXZ-F	CTACCTCGTGAAGAAGGTGAC
TRXZ-R	TAGAAATCCACGATGAGGGGC
psaA-F	GCGAGCAAATAAAACACCTTTC
psaA-R	GTACCAGCTTAACGTGGGGAG
psbA-F	CCCTCATTAGCAGATTCGTTT
psbA-R	ATGATTGTATTCCAGGCAGAGC
petA-F	TTAGCGAATAAGCCTGTG
petA-R	ATTGGGACGATAACTCTG

rbcL-F	CTTGGCAGCATTCCGAGTAA
rbcL-R	ACAACGGGCTCGATGTGATA
Lhcb1-F	GCAGTAGATGTACGTACGTGTA
Lhcb1-R	ACCTGCACAGAAATACACACTA
Lhcb2-F	TGTGTACACTTGTAGTAGCCAC
Lhcb2-R	CACACACACACACAATTAAGGT
Lhcb3-F	GATGGCGTCCACGATCAT
Lhcb3-R	GTACTTCACCCTATCCGGC
CRS2-F	CGCTGCTCGGAATAGGTT
CRS2-R	CCGTTGAAGACGCAATACAC
actin-F	CGGGAAATTGTGAGGGACATG
actin-R	AGGAAGGCTGGAAGAGGACC
CRS2-com-F	AGAGTCGACCTGCAGCGAAGTCGTAGCTATATATAGCTACCG
CRS2-com-R	CTTGCATGCCTGCAGGTAAACAATTTGTGTTCTTAAGAGCAAT
CRS2-GUS-F	AGAGTCGACCTGCAGCGAAGTCGTAGCTATATATAGCTACCG
CRS2-GUS-R	TCAGATCTACCATGGGGCGAGGGGAGAGGAGAAG
CRS2-GFP-F	ACAATTACAGTCGACATGTCGCTTGCCGTGGCC
CRS2-GFP-R	TCCTCTAGAGTCGACAACACTGTGGAACTTGTACTTCTGAAC
